# Understanding the Corrosion Behavior of Nickel–Aluminum Bronze Induced by Cavitation Corrosion Using Electrochemical Noise: Selective Phase Corrosion and Uniform Corrosion

**DOI:** 10.3390/ma16020669

**Published:** 2023-01-10

**Authors:** Liang Li, Yanxin Qiao, Lianmin Zhang, Aili Ma, Rongyao Ma, Yugui Zheng

**Affiliations:** 1 CAS Key Laboratory of Nuclear Materials and Safety Assessment, Institute of Metal Research, Chinese Academy of Sciences, Shenyang 110016, China; 2 School of Materials Science and Engineering, Jiangsu University of Science and Technology, Zhenjiang 212003, China

**Keywords:** cavitation erosion, nickel–aluminum bronze, selective phase corrosion, electrochemical noise

## Abstract

Nickel–aluminum bronze (NAB) is widely used to fabricate flow-handling components because of its good cavitation corrosion (CE) resistance and superior casting property. The existence of different phases, e.g., the α phase, β phase and κ phase, can cause significant selective phase corrosion on NAB. However, under the action of CE with different times, the influence of these phases on the corrosion behavior of NAB, including selective phase corrosion and uniform corrosion, needs to be further studied, which can contribute to a deep understanding of the CE mechanism of NAB in corrosive media. In this work, the corrosion behavior of NAB in 3.5 wt.% NaCl solution after different CE times was evaluated by electrochemical noise (EN), combined with scanning Kelvin probe force microscopy (SKPFM) and morphology analysis. The results showed that the corrosion behavior of NAB was closely associated with the variation in its complex microstructure after different CE times. Selective phase corrosion played a crucial role in the surface damage before 0.5 h of CE. With the prolongation of CE time, the stripping of κ phases decreased the degree of selective phase corrosion of NAB. As a result, both selective phase corrosion and uniform corrosion presented equal performances after 1 h of CE. However, after CE for 2–5 h, uniform corrosion had a dominant impact on the surface damage of NAB. Eventually, the corrosion mechanism of NAB after different CE times was clarified based on the relevant experimental results.

## 1. Introduction

Cavitation erosion (CE) refers to the failure form of surface damage that flow-handling components frequently encounter due to the collapse of bubbles in a hydrodynamic environment [1,2,3], especially for the flow-handling components used in marine environments, such as propellers, water turbines, pipes, etc. In corrosive media, flow-handling components often suffer from the combined interactions of mechanical impact and chemical corrosion. Mayer et al. [4] found that CE had a more remarkable influence on the corrosion kinetics of Cr3C2-25NiCr thermal spraying coating than the pure corrosion effect. Kwok et al. [5] studied the synergistic effect of CE and the corrosion of different engineering alloys, and the relevant results showed that pure corrosion had the smallest impact on the CE damage of copper alloys, while the synergistic effect of CE and corrosion played a crucial role for steel and cast iron. Qin et al. discovered that the increase in compressive stress enhanced the corrosion-induced CE effect of bronze, making it the dominant factor in the CE damage process [6]. However, the existing investigations rarely mention the effects of CE-induced surface damage on material corrosion behavior. The exploration in this aspect would be helpful for a more comprehensive and in-depth understanding of the CE damage mechanism of materials in corrosive media.

Nickel aluminum bronze (NAB) is widely used to fabricate flow-handling components because of its good CE resistance and superior casting property. Traditional NAB is mainly based on copper, and the added elements, including Ni, Al, Mn, Fe, etc., can enhance its mechanical properties or corrosion resistance [7,8]. For instance, Ni and Al can remarkably improve the corrosion resistance of bronze by forming a protective corrosion product film, Mn can endow bronze with a good work hardening ability, Fe can refine the grain size and improve the tensile strength of NAB. Generally, the structure and composition of cast NAB is very complex and include the copper-rich α phase, residual martensite β phase and four intermetallic compound κ phases, i.e., κ_I_, κ_II_, κ_III_ and κ_IV_ phases [9,10]. The κ phases are hard and precipitate in α phase matrices, which improves the CE resistance of the bronze. However, the non-uniform distribution of κ phases in α phase matrices increases the corrosion sensitivity of NAB.

The CE and corrosion behavior of NAB in simulated seawater have been widely investigated [7,11,12,13,14,15]. Luo et al. found that the mechanical effect played a major role in the material damage under the action of CE in 3.5 wt.% NaCl solution, while the corrosion effect was relatively small [12]. Zhang et al. reported that micro-cracks preferentially occurred at the boundaries of the κ/α phases in the process of CE, while the cracks easily laterally extended inside the α phase, due to the uniform distribution of the κ_IV_ phase [13]. The CE behavior of NAB was studied by Tian et al. in simulated seawater, and the results showed that the microcracks and selective phase corrosion together weakened the bonding force of the κ/α boundary, resulting in the exfoliation of the κ phases [15]. Meanwhile, in view of the differences in phase composition or structure, obvious selective phase corrosion of NAB appeared in the corrosion solution [8,16,17,18]. In addition, it was recorded by Wharton et al. that the crevice corrosion of NAB only existed in the α+κ_III_ eutectoid region, and the copper-rich α phases were preferentially attacked [17], which was consistent with the findings reported by other scholars [10,19,20]. Neodo et al. studied the effect of pH change on the selective phase corrosion of NAB in 3.5 wt.% NaCl solution and the κ phases preferentially dissolved when the pH < 4, while the α phase first dissolved when the pH > 4 [18]. Therefore, it is clear that the same phase can play different roles in the selective corrosion of NAB under different environmental conditions. Although the CE and corrosion behavior of NAB have been studied by many scholars, the corrosion behavior of NAB, including selective phase corrosion and uniform corrosion, induced by CE with different times has not been systematically investigated, since the conventional electrochemical methods, such as electrochemical impedance spectroscopy, linear polarization resistance and potentiodynamic polarization, cannot effectively detect the subtle changes caused by selective phase corrosion. Consequently, it is necessary to seek other methods to carry out relevant research.

Electrochemical noise (EN) technology has unique advantages in the investigation of local corrosion, especially in pitting corrosion, stress corrosion and selective phase corrosion [21,22,23,24,25,26]. EN can continuously monitor the corrosion behaviors of materials in unstable states caused by random non-equilibrium fluctuations in the electrode potential or external measured current. With the help of EN, Zhang et al. studied the corrosion behavior of AZ91D magnesium alloy in alkaline chloride solution, and they deeply analyzed the development process of pitting corrosion through the time domain and frequency domain of EN [23]. It was reported by Calabrese et al. that EN technology can identify electrochemical damage, such as pitting activation and stress corrosion cracking stabilization [25]. Based on the results of wavelet analysis, Li et al. found that for the appearance of selective phase corrosion, the cumulative distribution of relative energy is concentrated in a long time scale [26]. Therefore, EN can be a powerful tool to systematically study the selective corrosion behavior of different phases in NAB following different CE times.

Based on the above considerations, the corrosion response of NAB in 3.5 wt.% NaCl solution after different CE times was investigated by EN in this paper. The evolution of the surface morphology of NAB after different CE times was observed by scanning electron microscopy (SEM). EN data combined with mass loss and SKPFM analysis were used to evaluate the effect of surface damage after different CE times on the corrosion behavior of NAB. This research could shed light on the influence of CE-induced surface damage on the selective phase corrosion and uniform corrosion behavior of materials and contribute to a more comprehensive and in-depth understanding of the CE damage mechanism of NAB in corrosive media.

## 2. EN Analysis

### 2.1. Wavelet Analysis

Aballe et al. first applied wavelet analysis to the field of EN [27]. In recent years, wavelet analysis of EN data has been widely used in the corrosion field. In this experiment, the EN signal is divided into smooth coefficient crystals (s8) and detail coefficient crystals (d1–d8) by eight-layer wavelet decomposition. The two kinds of wavelet coefficients represent the overall trend and local trend of corrosion events, respectively. More details on wavelet analysis can be obtained by referring to the literature of other scholars [27,28,29]. The relative energy fraction of each crystal can be calculated by Equation (1), and the results of the wavelet transform are shown by the energy distribution plot (EDP).

(1) $$E_{d}^{j}=\frac{1}{E}\sum_{n=1}^{N/2^{j}}d_{j,\;n}^{2}\;j=(1,2,3,\ldots \ldots 8)$$

### 2.2. Shot Noise Theory

According to the theory of shot noise, the current noise signal is composed of charge packets that deviate from the baseline [30,31]. In the corrosion process, the current is carried by discrete carriers, which will produce shot noise. The movement of discrete carriers is a relatively independent and random process [32]. Therefore, two characteristic parameters, *f*_n_ and *q,* can be extracted from the shot noise signal of corrosion. The parameters *f*_n_ and *q* can be calculated according to Equations (2) and (3), which are as follows:

(2) $$fn=\frac{{\bar{I}}_{\mathrm{corr}}}{q}=\frac{B^{2}}{\psi_{E}A}$$

(3) $$q=\frac{\sqrt{\psi_{E}\psi_{I}}}{B}$$

where *B* is the Stern–Geary constant, *A* is the electrode area, *ψ*_E_ and *ψ*_I_ are the power spectral densities of the potential noise and the current noise at 0.01 Hz, respectively.

### 2.3. Corrosion Initiation Rate Based on Shot Noise

Weibull analysis is an analysis method based on a stochastic model, which is usually used for life prediction and reliability analysis [33]. It can quantitatively analyze data, even if two or more failure modes exist at the same time [34]. In order to obtain the cumulative probability *F*(*f*_n_), all the *f*_n_ data are arranged in ascending order and *F*(*f*_n_) is calculated as M/(N + 1), where M and N are the order and the total number of *f*_n_ data, respectively. The cumulative probability of the failure system can be calculated based on Equation (4), which is as follows:

(4) $$F\left(t\right)=1-\exp\left(-\frac{t^{m}}{n}\right)$$

Equation (4) can be rearranged into Equation (5), as follows:

(5) $$\ln\left\{\ln\frac{1}{1-F\left(t\right)}\right\}=m\ln\left(t\right)-\ln\left(n\right)$$

(6) $$r(t)=\frac{m}{n}t^{m-1}$$

where *m* and *n* are the shape and the scale parameters, respectively, which are decided by the slope and the intercept of the linear area of the graph plotted by Equation (5). The incubation rate *r*(*t*) of corrosion events can be obtained by substituting m and n into Equation (6).

### 2.4. Corrosion Growth Probability Based on Shot Noise

Gumbel analysis can predict the maximum corrosion growth probability by introducing extreme value statistics theory to simulate the growth behavior of pits [35]. The specific operation method is as follows: all the *q* values are arranged in descending order, and the cumulative probability *F*(*Y*) of the decreasing variable *Y* is obtained by Equation (7). Then, *q* is taken as the abscissa and *Y* as the ordinate to obtain the Gumbel distribution plots. The variables of *a*, *b* are the slope and the intercept of the linear region of the Gumbel distribution plots, respectively. Then, the parameters of *a*, *b* can be substituted into Equations (8) and (9) to obtain the scale parameter α and the location parameter *μ*. The growth probability of corrosion (*P*_c_) can be calculated by Equation (10).

(7) $$F(Y)=1-\frac{M}{N+1}$$

(8) a=1/b

(9) μ=−a/b

(10) $$P_{c}=1-\exp\left\{-\exp\left[-\frac{\left(q-\mu \right)}{\alpha }\right]\right\}$$

## 3. Experimental Section

**Figure 1:**
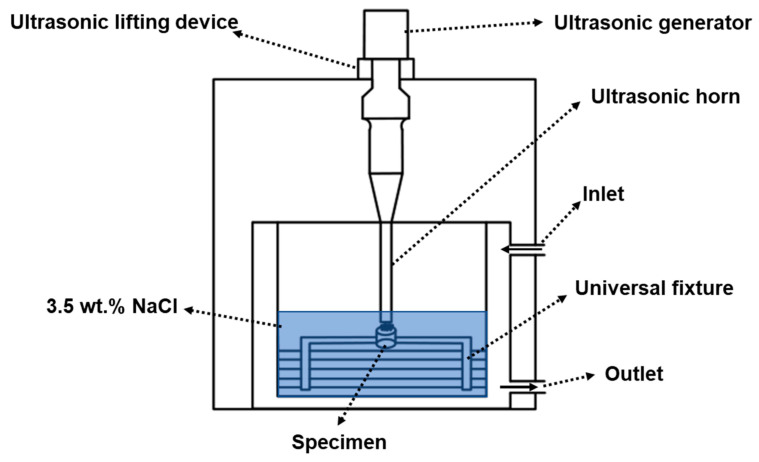
Schematic diagram of the CE equipment.

The chemical composition of the NAB used for the experiment is shown in Table 1. According to the ASTM Standard G32-10 [36], CE tests were conducted using an ultrasonic CE device (Nanjing Xianou Instrument Manufacturing Co., Ltd, Nanjing, China), which worked at a frequency of 20 kHz, an output power of 2.5 kW and a peak-to-peak amplitude of 60 μm. The schematic diagram of the device is shown in Figure 1. The size and shape of the samples used in the experiment can be found in previous studies [37]. Prior to the CE tests, the samples were ground to 400, 800, 2000 and 5000 grits in sequence by SiC sandpapers, polished with 0.5 μm diamond paste, then degreased with absolute ethanol and dried with cold air. The samples were placed 0.05 mm below the cavitation horn in 3.5 wt.% NaCl solution. In addition, the experimental temperature was maintained at 25 ± 2 °C by a temperature control box. The weight of the samples was measured using an analytical balance with a sensitivity of 0.1 mg. At least three parallel experiments were conducted to ensure the accuracy of the data.

The EN test was performed in a Faraday cage using a three-electrode system, including two working electrodes of equal samples and a saturated calomel electrode (SCE). The EN data were collected by the ES410 software (GamrySoftware_7.8.1.7232), and its software setting parameters included a sampling frequency of 5 Hz, a test time of 5 h and an interval of 1024 points. The EN data collected in the experiment were DC denoised by the 5th order polynomial, while the Sym8 wavelet was used for wavelet analysis. All the noise data were processed by MATLAB. The morphologies and the roughness of the samples were characterized by scanning electron microscopy (SEM, INSPECT F50, FEI Company, Hillsboro, OR, USA) and by a white light interferometer (Micro XAM 3D, KLA Corporation, Chandler, AZ, USA), respectively. In addition, the microscopic height and the potential difference of each phase prior to CE were evaluated by atomic force microscopy (AFM, Bruker Corporation, Billerica, CA, USA) and scanning Kelvin probe force microscopy (SKPFM, Bruker Corporation, Billerica, CA, USA).

## 4. Results and Discussion

### 4.1. Mass Loss

**Figure 2:**
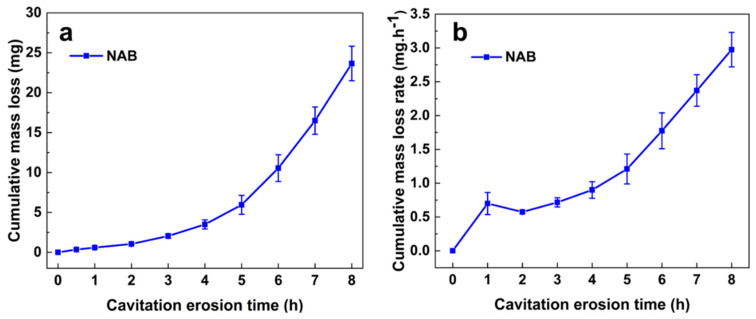
Cumulative mass loss (**a**) and cumulative mass loss rate (**b**) of NAB after different CE times.

Figure 2a displays the relationship between the cumulative mass loss of NAB and time change. The results show that the cumulative mass loss of NAB increases as the CE time increases. After CE for 8 h, the cumulative mass loss of NAB in 3.5 wt.% NaCl solution reaches 23.65 mg. The variation in the cumulative mass loss rate versus CE time is shown in Figure 2b. It is interesting to note that the mass loss rate increases sharply at the initial stage of CE (before 1 h). The sharp increase in the cumulative mass loss rate might be attributed to the shedding of the hard κ phases on the surface of NAB, which was also reported by Tian et al. [15]. Afterwards, the cumulative mass loss rate continuously increases as the CE time is extended. The change in cumulative mass loss clearly shows that the incubation period is 1 h for NAB in 3.5 wt.% NaCl solution, which is consistent with the results of other studies [7,13].

### 4.2. Evolution of CE Morphology and Surface Roughness

**Figure 3:**
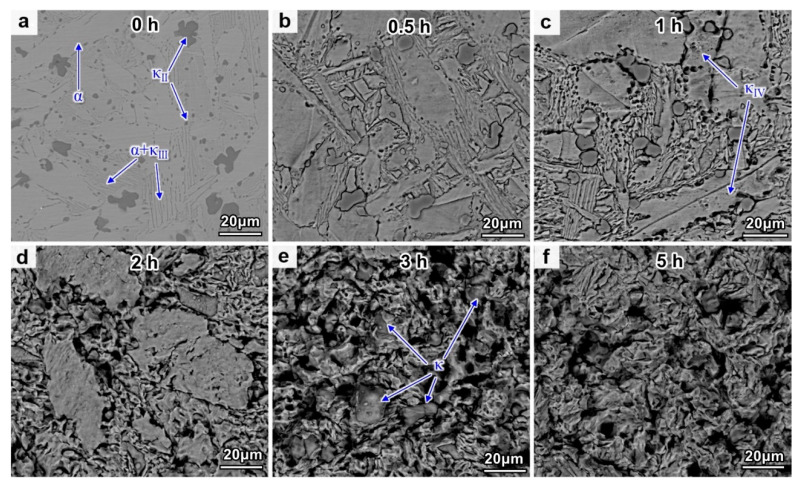
Surface morphologies of NAB after different CE times: (**a**) 0 h; (**b**) 0.5 h; (**c**) 1 h; (**d**) 2 h; (**e**) 3 h; (**f**) 5 h.

The morphological evolution of NAB after different CE times is shown in Figure 3. The original microstructure is shown in Figure 3a. The κ_II_ phase is globular or rosette-shaped and is generally distributed at the boundaries of α phases. The κ_III_ phase in the eutectoid has a lamellar structure, while the κ_IV_ phase is not observed before CE, due to instrument limitation. The hard κ phase has a higher resistance to CE impact, compared with the α phase. Meanwhile, the κ phase usually acts as the cathode and the α phase often acts as the anode in 3.5 wt.% NaCl solution, thereby the corrosion of the α phase will be accelerated when the two kinds of phases are in contact with one another. Therefore, in the early stage of the CE process, the CE damage is mainly attributed to the damage of the α phase in theory. However, the peeling of the small-sized κ_II_ phase can be clearly observed after CE for 0.5 h and 1 h (Figure 3b), and the honeycomb pits in the α phase after 1 h of CE prove the existence of the κ_IV_ phase (Figure 3c). A large number of holes and micro-cracks appear at the phase boundary of α and κ. The exfoliation of these hard phases is mainly related to the following two factors. Firstly, selective phase corrosion reduces the binding force between the κ phases and the surrounding α phase matrix [18]. Secondly, the CE-induced stress concentration at the α/κ phase boundary causes the formation of cracks, which propagate and promote the peeling of the κ phases [15]. After CE for 2 h, the surface of the sample is severely damaged, and only some residual α phases can be observed on the surface (Figure 3d). However, the residual α phase is completely stripped after CE for 3 h, leaving the internal κ phases extruded on the surface (Figure 3e). After CE for 5 h, these κ phases are further peeled off and the entire surface is honeycombed (Figure 3f).

**Figure 4:**
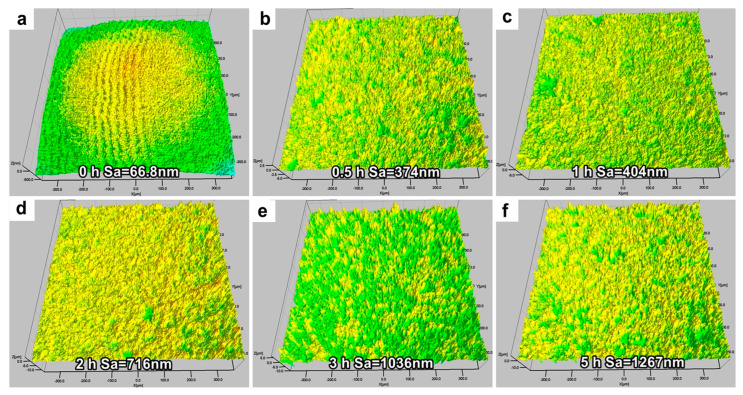
Surface roughness of NAB after different CE times: (**a**) 0 h; (**b**) 0.5 h; (**c**) 1 h; (**d**) 2 h; (**e**) 3 h; (**f**) 5 h.

Figure 4 depicts the surface roughness (*S*_a_) of the NAB after different CE times. The result demonstrates that the *S*_a_ value increases as the CE time increases. The *S*_a_ of the sample prior to CE is 66.8 nm. The *S*_a_ value significantly increases after 0.5 h of CE, and this is attributed to the peeling of these small κ phases. The value of *S*_a_ increases slowly after 3 h of CE, because the presence of κ phases inhibits the longitudinal propagation of the cracks and facilitates the uniform exfoliation of the sample’s surface.

### 4.3. AFM and SKPFM Analysis

**Figure 5:**
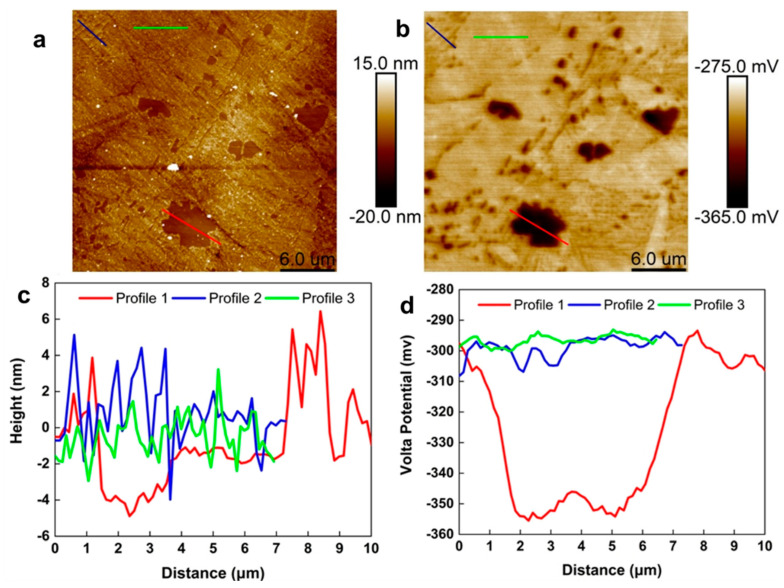
AFM topography image of the NAB (**a**), Volta potential map of NAB (**b**), the height profiles (**c**) and the Volta potential profile (**d**) of the κ_II_ phase, κ_III_ phase and α phase.

Figure 5a,b show the topographic image and the Volta potential distribution map of the NAB, respectively. The brightness difference in the different areas of the two figures matches the height and Volta potential of the sample surface, respectively. Therefore, a brighter area indicates a greater height and Volta potential. The κ phases demonstrate lower heights and Volta potential than those of the α phases. It is noteworthy that the κ_IV_ phase remains in the α phase and cannot be detected by the instrument. Figure 5c,d show the line profiles of the height and the Volta potential. The results show that the κ_II_ phase presents the lowest height, which is 5 nm away from the polished surface. The height of the κ_III_ and α phases is relatively close, possibly because they are adjacent to each other in the eutectic microstructure. In addition, the polishing methods and the etchant can severely affect the Volta potential results [38,39]. The difference between the Volta potential of a certain phase and the α matrix can be expressed as Δ*E*. As shown in Figure 5d, the Δ*E* between the α phase and the κ_II_ phase reaches about 57 mV. The Δ*E* result follows the following order: ∆*E*_α_ ˂ ∆*E*_κIII_ ˂ ∆*E*_κII_. Based on the above results and relevant literature [10,15], it can be concluded that the boundary of the α/κ_II_ phase demonstrates the most severe selective phase corrosion in 3.5 wt.% NaCl solution, followed by the boundary of the α/κ_III_ phase.

### 4.4. Electrochemical Noise

**Figure 6:**
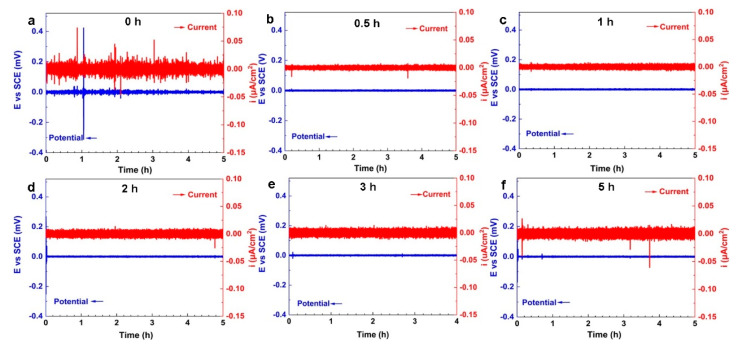
The electrochemical potential noise (EPN) and electrochemical current noise (ECN) signals of NAB after different CE times: (**a**) 0 h; (**b**) 0.5 h; (**c**) 1 h; (**d**) 2 h; (**e**) 3 h; (**f**) 5 h.

Figure 6 presents the electrochemical potential noise (EPN) and electrochemical current noise (ECN) signals of NAB after different CE times. It is well known that the corrosion characteristics of electrode surfaces, such as corrosion type and corrosion intensity, can be determined according to the amplitude and fluctuation of EN signals [40,41]. The EPN and ECN signals of NAB prior to CE show a large number of transient peaks over a long time period, indicating the characteristics of localized corrosion. These signals are generated by selective phase corrosion between the κ phase and the α phase. With the extension of CE time, the characteristic signal of localized corrosion gradually decreases. The transient amplitude of EPN noise increases with the prolongation of CE time, implying an increase in surface activity and the deterioration of the corrosion resistance of the sample.

**Figure 7:**
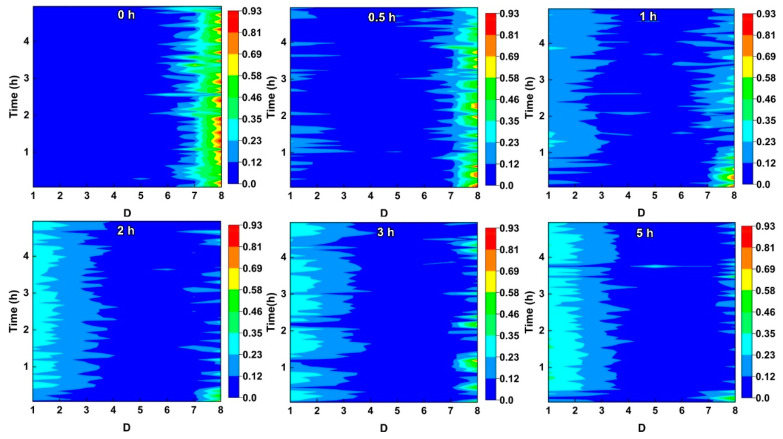
Energy distribution plots (EDP) for the current noise of NAB after different CE times.

The ECN data of NAB recorded after different CE times are converted into energy distribution plots (EDP) through wavelet analysis, as shown in Figure 7. It is well documented that the fast processes of corrosion events usually correspond to a short time scale, while the slow reactions often correspond to a long time scale [28,42]. In Figure 7, the time scale of crystal *d* gradually increases from d1 to d8. The relative energies in crystals d1–d3, d4–d6, and d6–d8 correspond to uniform corrosion (passivation), metastable pitting growth, and stable pitting growth or diffusion control, respectively. For the NAB without CE, the relative energy is mainly distributed in the crystals d6–d8. These may be associated with a large number of localized corrosion signals that are similar to stable pitting corrosion. These localized corrosion signals are attributed to preferential corrosion at the α/κ phase boundary. After CE for 0.5 h, a small amount of relative energy is distributed in d1–d3, which may be induced by the weakening of selective phase corrosion and the strengthening of uniform corrosion. For the samples after CE for 1 h, the relative energy is uniformly distributed in crystals d1–d3 and crystals d6–d8, implying that the uniform corrosion and selective phase corrosion are similar to each other. With the extension of CE time, the relative energy is mainly distributed in the crystals d1–d3 after CE for 2–5 h, which means that the sample is mainly subjected to uniform corrosion. It should be noted that the role of selective phase corrosion slightly intensifies after 3 h of CE, which may be attributed to the reappearance of the internal κ phases under the action of CE.

**Figure 8:**
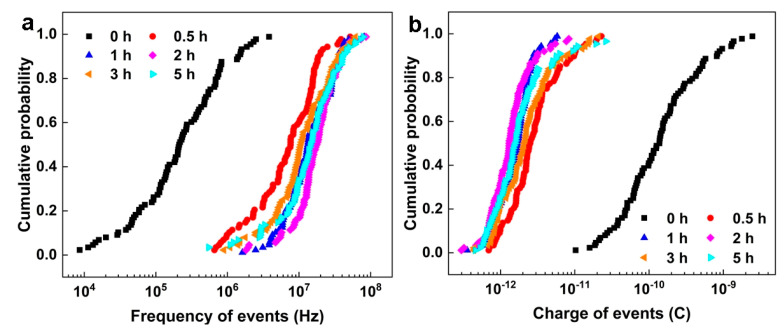
Cumulative distribution of *f*_n_ (**a**) and *q* (**b**) of NAB after different CE times.

Figure 8 shows the event frequency (*f*_n_) and corrosion charge (*q*) of NAB after different CE times. Generally, the increase in *f*_n_ indicates that the sample is more inclined to uniform corrosion, whereas the decrease in *f*_n_ implies that the corrosion mechanism favors localized corrosion. The increasing *q* value indicates that the charge released by the corrosion event is increased, and the corrosion mechanism is more inclined to localized corrosion, while the decrease in *q* suggests that the corrosion mechanism favors uniform corrosion [43,44,45]. Compared with the sample without CE, the *f*_n_ of the sample after CE for 0.5 h, 1 h and 2 h continuously increases and *q* continuously decreases, which indicates that the localized corrosion signal becomes weak and the signal of uniform corrosion is enhanced. After CE for 3 h, the *f*_n_ of the samples decreases and *q* increases, showing that the tendency of localized corrosion is slightly increased. However, the samples are dominated by uniform corrosion after 5 h of CE. The EN signal provided by shot noise is limited, which will be discussed in detail later.

**Figure 9:**
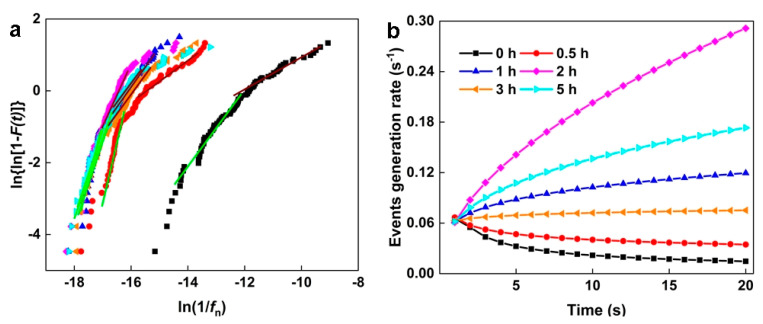
(**a**) Weibull distribution of *f*_n_ of NAB after different CE times: (**b**) Plots of the incubation rate of selective phase corrosion after different CE times.

Figure 9a shows the Weibull distribution of NAB relative to ln(1/*f*_n_) after different CE times. The two linear regions indicate two failure modes, which depend on the size of *f*_n_. In the EN test, only uniform corrosion and selective phase corrosion with localized corrosion characteristics occurred. Uniform corrosion generally has a larger *f*_n_, while localized corrosion usually has a smaller *f*_n_ [30,43,46]. Therefore, the linear area on the left side of Figure 9a represents uniform corrosion, while the linear area on the right side corresponds to selective phase corrosion. The shape parameter (m) and scale parameter (n) are obtained by fitting the low-frequency linear region. It is substituted into Equation (6) to calculate the incubation rate of selective phase corrosion with localized corrosion characteristics (Figure 9b). The results show that the incubation rate of selective phase corrosion gradually increases after CE for 0.5–2 h, compared with the samples without CE. However, the incubation rate of selective phase corrosion gradually declines after 3 h of CE and increases again after 5 h of CE. These changes can be related to the defects induced by CE on the sample’s surface.

**Figure 10:**
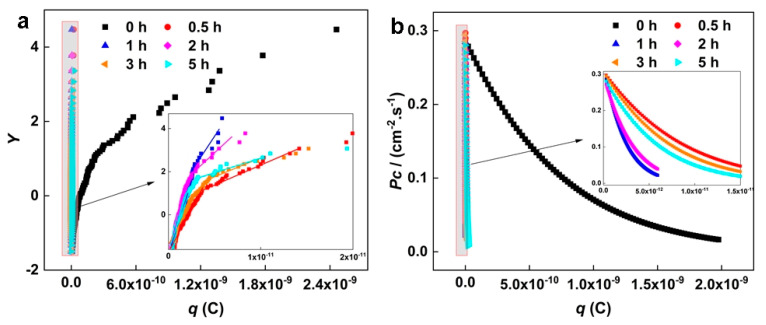
(**a**) Gumbel distribution curve of *q* of NAB after different CE times: (**b**) Plots of the growth probability of selective phase corrosion after different CE times.

The Gumbel distribution function with corrosion charge *q* can predict the maximum corrosion charge of NAB after different CE times. Figure 10a presents the corrosion charge *q* of NAB after different CE times. The linear region with a larger *q* value indicates selective phase corrosion. On the contrary, the linear part with a smaller *q* value implies uniform corrosion. The scale parameter α and the location parameter μ are obtained by fitting the linear region with a high *q* value, which are substituted into Equation (10) to calculate the corrosion growth probability *p*_c_. As shown in Figure 10b, it is obvious that the sample without CE has the maximum corrosion growth probability, with respect to selective phase corrosion. For the samples after 0.5 h and 1 h of CE, the accelerated growth probability of selective phase corrosion significantly decreases. After CE for 2 h and 3 h, the growth probability of selective phase corrosion slightly increases, then decreases again after 5 h of CE.

Based on the results of shot noise, for the NAB sample without CE, the selective phase corrosion with localized corrosion characteristics has the minimum incubation rate and the maximum corrosion growth probability, indicating remarkable selective phase corrosion. This is mainly attributed to the large amount of κ phases, which promote the dissolution of the boundary of the α/κ phase. After CE for 0.5 h and 1 h, the incubation rate of selective phase corrosion increases and the growth probability of selective phase corrosion decreases, suggesting that the role of selective phase corrosion is weakened. This change may be due to the shedding of small κ phases. For the sample after 2 h of CE, the incubation rate of selective phase corrosion further increases and the selective phase corrosion growth probability increases, indicating that more local corrosion signals appear on the sample’s surface. This phenomenon may be attributed to the blocking cell effect on porous surfaces, which generates local corrosion signals, except for selective phase corrosion [47]. For the sample after CE for 3 h, the incubation rate of selective phase corrosion decreases and the growth probability of selective phase corrosion increases, compared with the sample with CE for 2 h, which implies that the role of selective phase corrosion is further enhanced. It is very likely that the κ phases inside the sample are exposed to the surface again, promoting the accelerated dissolution of the boundary of the α/κ phase. After CE for 5 h, the incubation rate of selective phase corrosion increases again and the selective phase corrosion growth probability decreases, which may be due to the further peeling of the internal κ phases and the weakened role of selective phase corrosion.

### 4.5. Corrosion Mechanism of NAB after Different CE Times

**Figure 11:**
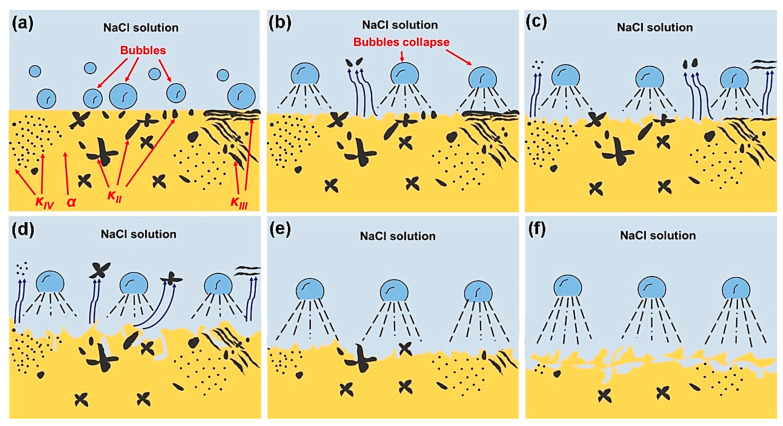
Corrosion mechanism diagram of NAB with different CE times: (**a**) CE bubbles appear on the surface of NAB with a complex phase structure; (**b**) the bubbles destroy the α phase and the small-sized κ_II_ phase peels off; (**c**) bubbles further destroy the boundary of the α/κ phase, causing the peeling of κ_II_, κ_III_ and κ_IV_; (**d**) the surface of the α phase is damaged by the bubbles, and the large-sized κ_II_ phase peels off; (**e**) the internal κ phases are exposed to the surface again; (**f**) the internal κ phases peel off again caused by the destruction of bubbles.

Based on the above research, it can be deduced that the corrosion behavior of NAB after different CE times is mainly influenced by the changed microstructure. The results of SKPFM show that a potential difference between the κ phase and the α phase exists, which explains the reason for the selective phase corrosion of the boundary of the α/κ phase in 3.5 wt.% NaCl solution. Furthermore, the damage mechanism diagram of NAB with different CE times is depicted in Figure 11, and the detailed explanation of the corrosion behavior of the NAB is described in detail by combining the results with morphology and noise data analysis. The selective phase corrosion occurs on the sample’s surface prior to CE, because a large number of κ phases precipitate inside the α phase matrix, accelerating the dissolution of the boundary of the α/κ phase (Figure 11a). After CE for 0.5 h, selective phase corrosion still plays a major role. However, the exfoliation of these κ_II_ phases significantly weakens the role of selective phase corrosion, and uniform corrosion gradually has an important impact on the corrosion of NAB (Figure 11b). After CE for 1 h, the further peeling of the small-sized κ phases occurs and uniform corrosion appears on the α phase (Figure 11c). As a result, selective phase dissolution and uniform corrosion demonstrate equal performances. For the sample after CE for 2–5 h, the surface of the sample mainly undergoes uniform corrosion. The large-sized κ_II_ phases on the sample’s surface are further peeled off after CE for 2 h. In addition, the stripping of the κ phase produces a large number of holes, which promotes the appearance of local corrosion signals and these signals are different from those associated with selective phase corrosion (Figure 11d). With the prolongation of CE time, the remaining α phase fragments are further removed after 3 h of CE and the internal κ phases are exposed again on the surface. As a result, selective phase corrosion is enhanced (Figure 11e). However, the exposed κ phases are also stripped after 5 h of CE, resulting in weakened selective phase corrosion (Figure 11f).

## 5. Conclusions

In this paper, the effect of CE-induced surface damage on the corrosion behavior of NAB, including selective phase corrosion and uniform corrosion, with different CE times was investigated by EN, combined with SKPFM and morphology analysis. The main conclusions are as follows:

1. The corrosion behavior of the NAB after different CE times was closely related to the change in microstructure. The essence of selective phase corrosion involved the galvanic corrosion between the α phase and the κ phase, which led to the corrosion along the boundary of the α/κ phase in 3.5 wt.% NaCl solution.
2. In the CE process, the hard κ phase had a higher resistance to CE impact compared with the α phase. Meanwhile, the κ phase often acted as the cathode and the α phase often acted as the anode, which caused the accelerated corrosion of the α phase when the two kinds of the phases were in contact with one another. Therefore, the damage to the κ phase was far less than that of the α phase. However, the stress concentration and selective phase corrosion indicated that cracks preferentially occurred at the boundary of the α/κ phase, and the κ phase with a small size was preferentially peeled off in the CE process.
3. Before CE for 0.5 h, selective phase corrosion played a leading role in the surface damage, which was mainly attributed to the existence of a large number of κ phases, accelerating the corrosion of the boundary of the α/κ phase due to the potential difference. With the prolongation of CE time, the effect of selective phase corrosion on the sample surface gradually declined, while the effect of uniform corrosion gradually increased. Both selective phase corrosion and uniform corrosion presented equal performances after 1 h of CE. The sample surface mainly demonstrated uniform corrosion after CE for 2 h–5 h. However, it is worth noting that the role of selective phase corrosion was enhanced after 3 h of CE, which could be caused by the accelerated selective phase corrosion, due to the exposure of the internal κ phase to the sample surface. A new corrosion mechanism of NAB after different CE times was revealed based on the relevant experimental results.

## Figures and Tables

**Table 1:** Chemical composition (wt.%) of the investigated NAB.

Brand	Al	Ni	Fe	Mn	C	Si	Cu
ZCuAl9Fe4Ni4Mn2	9.66	4.66	4.45	2.19	0.05	0.10	Bal.

## Data Availability

The raw and the processed data are available from L. M. Zhang (Email: lmzhang14s@imr.ac.cn) upon reasonable request.
